# Novel Pullulan/Gellan Gum Bilayer Film as a Vehicle for Silibinin-Loaded Nanocapsules in the Topical Treatment of Atopic Dermatitis

**DOI:** 10.3390/pharmaceutics14112352

**Published:** 2022-10-31

**Authors:** Mailine Gehrcke, Carolina Cristóvão Martins, Taíne de Bastos Brum, Lucas Saldanha da Rosa, Cristiane Luchese, Ethel Antunes Wilhelm, Fabio Zovico Maxnuck Soares, Letícia Cruz

**Affiliations:** 1 Laboratório de Tecnologia Farmacêutica, Programa de Pós-Graduação em Ciências Farmacêuticas, Centro de Ciências da Saúde, Universidade Federal de Santa Maria, Santa Maria 97105-900, RS, Brazil; 2 Laboratório de Pesquisa em Farmacologia Bioquímica—Centro de Ciências Químicas, Farmacêuticas e de Alimentos, Universidade Federal de Pelotas, Pelotas 96010-900, RS, Brazil; 3 Laboratório de Biomateriais, Centro de Ciências da Saúde, Departamento de Odontologia Restauradora, Universidade Federal de Santa Maria, Santa Maria 97015-372, RS, Brazil

**Keywords:** nanocapsules, films, silibinin, pullulan, gellan gum, atopic dermatitis

## Abstract

In this study a novel gellan gum/pullulan bilayer film containing silibinin-loaded nanocapsules was developed for topical treatment of atopic dermatitis (AD). The bilayer films were produced by applying a pullulan layer on a gellan gum layer incorporated with silibinin nanocapsules by two-step solvent casting method. The bilayer formation was confirmed by microscopic analysis. In vitro studies showed that pullulan imparts bioadhesitvity for the films and the presence of nanocapsules increased their occlusion factor almost 2-fold. Besides, the nano-based film presented a slow silibinin release and high affinity for cutaneous tissue. Moreover, this film presented high scavenger capacity and non-hemolytic property. In the in vivo study, interestingly, the treatments with vehicle film attenuated the scratching behavior and the ear edema in mice induced by 2,4-dinitrochlorobenzene (DNCB). However, the nano-based film containing silibinin modulated the inflammatory and oxidative parameters in a similar or more pronounced way than silibinin solution and vehicle film, as well as than hydrocortisone, a classical treatment of AD. In conclusion, these data suggest that itself gellan gum/pullulan bilayer film might attenuate the effects induced by DNCB, acting together with silibinin-loaded nanocapsules, which protected the skin from oxidative damage, improving the therapeutic effect in this AD-model.

## 1. Introduction

Atopic dermatitis (AD), a chronic inflammatory skin condition, results from a complex interaction among the genetic predisposition, the environmental factors, as well as the dysfunctions in the skin barrier and in the permeability of allergens and pathogens agents [1]. These pathological mechanisms could play synergistically to maintain the clinical symptoms of AD, including pruritus, eczematous lesions, remodeling of the skin surface and generalized skin dryness due to the constant inflammation [2]. The current treatments for AD are based on skin barrier recovery and the use of corticosteroids and topical and systemic immunosuppressant, which are used as unique or combined therapy. However, these pharmacological treatments present limited effectiveness and relevant side effects [3].

In this context, the attempts to planning and developing a new, safe, and effective therapeutic strategy for AD have been receiving notable priority. Several studies have been demonstrated the safety and the effectiveness of naturally occurring substances in the AD treatment and thereby, their arise as an interesting option to overcome conventional issues of the available pharmacological treatment [4]. Silibinin (SB), the main biologically active flavonoid extracted from *Silybum marianum* seeds, is well recognized for its potent antioxidant and anti-inflammatory properties [5]. This flavonoid has been widely studied to prevent and to treat inflammatory skin disorders, including dermatoses [6,7,8]. However, SB is poor soluble in water and its solubilization in others solvents is limited [9]. Few studies have explored the development of formulations that allow the application of this flavonoid on the skin. The existent studies involve the use of nanotechnology to improve the SB solubility and performance against skin diseases [7,8,10].

Moreover, drug delivery systems based on nanotechnology have shown a promising strategy for AD management due to the nanostructures’ properties to achieve enhanced skin penetration and retention, release control, and reduced side effects [11,12,13]. Among these nanostructured systems stand out the nanocapsules (NC) which have a core/shell structure constituted by oil and polymer, respectively [14]. This structural organization favors the encapsulation of lipophilic substances into NC, increasing the solubility and therapeutic efficacy of these substances [7,10,15].

In this same way, polymeric films present many advantages to treat inflammatory skin diseases, such as AD, due to their potential to adhere to the skin providing protection and hydration for this tissue [16,17,18]. For the films development aiming cutaneous use, natural polysaccharides have shown to be promising candidates due to low toxicity, biocompatibility and biodegradability [19]. Among such polysaccharides is gellan gum, which has been shown to be suitable for the formation of ultrathin films with physical and mechanical resistance and with swelling capacity, being promising for protecting the area and absorbing exudate of lesions [20,21]. Another natural polysaccharide that has attracted the interest of researchers is pullulan, which forms elastic, bioadhesive and fast-dissolving films [18,22]. In addition, it was suggested by Jeong and co-workers that pullulan may present a physical action against AD [16].

Studies have reported the bilayer films as an advantageous alternative to polymeric blends for cutaneous delivery. These films are able to preserve the intrinsic characteristics of each film-forming agent in it respective polymeric layer, improving the film properties [23,24]. Besides, combining nanostructures into polymeric films for cutaneous delivery renders a dosage form that can be administered over the affected skin, promoting a physical barrier for injured skin, and allowing a lower frequency of administration, which improves the therapeutic efficacy [25,26].

Also, NC have shown a promising alternative to incorporate lipophilic substances into hydrophilic films [18]. In a previous study, we demonstrate the promising advantages of SB encapsulation into NCs followed by their incorporation into gellan gum films for cutaneous delivery. The developed films presented physical and mechanical resistance and fluid absorption capacity. Besides, due to the high SB lipophilicity, the nanoencapsulation was fundamental to guarantee it dosage homogeneity into gellan gum film. Also, the NC association into the gellan gum film provided increased SB stability, as well as controlled release and improved skin permeation to the dermis [27]. These results plus to the attractive pullulan films characteristics stimulated our curiosity about combining them in a novel bilayer film to treat AD.

The objective of the present study was to develop a bilayer film composed by gellan gum layer incorporated with SB-loaded NC as the bottom side and with a pullulan layer as the top side, as well as to evaluate the film potential against AD-like skin lesions. This novel nano-based bilayer film was engineered such that the distinct layers provide their respective beneficial features for AD treatment.

## 2. Materials and Methods

### 2.1. Materials

SB (98% purity), Span^®^ 80 (sorbitan monooleate) were obtained from Sigma Aldrich (São Paulo, SP, Brazil). Ethylcelullose was donated by Colorcon (Cotia, SP, Brazil). Medium chain triglycerides and Tween^®^ 80 (polysorbate 80) were bought from Delaware (Porto Alegre, RS, Brazil). Gellan gum (Kelcogel^®^) and pullulan were donated by CP Kelco (Limeira, RS, Brazil) and Hayashibara, respectively. 2,4-dinitrochlorobenzene (DNCB) was purchased from Sigma (St. Louis, MO, USA) and it was used as an inductor of atopic dermatitis (AD)-like lesions. The hydrocortisone ointment (HC) was obtained commercially and it was used as a reference drug. All other solvents and reagents were analytical grade and used as received.

### 2.2. Methods

#### 2.2.1. Preparation of Nanocapsule Suspensions and Bilayer Films

SB-loaded NC were prepared by the interfacial deposition of preformed polymer method [28], at a concentration of 2.5 mg/mL as described previously [27]. The organic phase was composed by SB (0.025 g), ethylcellulose (0.25 g), Span^®^ 80 (0.1925 g), medium chain triglycerides (0.75 g) and acetone (68 mL). The aqueous phase was composed of water (132 mL) and Tween^®^ 80 (0.1925 g). Then, the organic phase was added under magnetic stirring into aqueous phase followed by solvent evaporation under reduced pressure to achieve a 10 mL final volume. Unloaded NC suspensions were prepared using this same protocol, omitting SB. After preparation, NC suspensions were characterized in terms of particle size and polydispersity index by photon correlation spectroscopy (1:500 dilution in ultrapure water), and zeta potential by the microelectrophoresis technique (1:500 dilution in 10 mM NaCl). These analyzes were conducted using the Zetasizer Nanoseries^®^ equipment (Malvern Instruments, Malvern, Worcestershire, UK). Besides, the total SB content in the suspension was assessed by its extraction from the nanocapsules using methanol and sonication (5 min), followed by high performance liquid chromatography analysis.

The bilayer films were produced by two-step solvent casting method. For this, firstly, the gellan gum dispersion was prepared by dispersing 0.25 g of this gum in 15 mL distilled water while heating at 80 °C, under magnetic stirring for 2 h. After, an amount of glycerol (1 g) was added to this dispersion. Subsequently, the mixture was removed from the heating and 10 mL of water or NC suspension were added to gellan gum dispersion to produce the first layer of the vehicle film or nano-based films, respectively. After, this mixture was instantly poured into a Petri dish (90 × 13 mm) and was partially dried at 40 °C for 15 h. Then, a water solution containing 3% (*w*/*v*) of pullulan and 0.5% (*w*/*v*) of glycerol was prepared at room temperature and under magnetic stirring for 30 min. After complete pullulan solubilization, this solution was poured on the surface of the first layer and dried at 40 °C for 24 h. The bilayer films were named BF NC SB, BF NC B and BF vehicle for films produced with nanoencapsulated SB, unloaded NC and control film, respectively.

#### 2.2.2. Scanning Electron Microscopy

The structure of the bilayer films was evaluated by scanning electron microscopy (SEM) (JEOL JSM 6360, Akishima, Japan). To visualize the layers, the films were cryofractured in order to analyze the sides sections after fracture. To carry out the analysis, the samples were previously placed on a double–sided adhesive carbon tape, mounted on the sample slab and coated with gold (Denton Vaccum II, 100 Ǻ, Moorestown, EUA) under reduced pressure. The samples were subsequently analyzed using an accelerating voltage of 10 kV. This analysis was also performed for a monolayer vehicle film (MF vehicle) which was produced containing only the layer of gellan gum and served as a control.

#### 2.2.3. Bilayer Films Characterization

The bilayer films were characterized by homogeneity of SB content, thickness, moisture, nanometric size maintenance and swelling index. For thickness measurement, films (*n* = 3) were prepared for each formulation and then five measurements were performed on each film (four in the corner and one in the middle). Mean thickness values were calculated and expressed in μm. For homogeneity of SB content, the films (*n* = 3) were cut into three fragments of 1 cm × 1 cm each. The SB content in each fragment was quantified by extracting the phytochemical in methanol, subjecting it to stirring for 20 min followed by sonication for another 20 min. Samples were filtered (0.45 μm) and analyzed by high performance liquid chromatography (HPLC), using a guard column and a Kinetex C_18_ Phenomenex column (250 mm × 4.60 mm, 5 μm; 110 Å) at room temperature. The mobile phase consisted of water pH 3.5 and acetonitrile (60:40, *v*/*v*) at isocratic flow rate (1.0 mL/min) and the detection wavelength used for SB was 288 nm, as described previously [27]. The mean values of the SB content were calculated and expressed in μg/cm^2^, and the content (%) was calculated in relation to the theoretical amount of SB present in the film.

The particle size and the polydispersity index of the NC after their incorporation into films were evaluated by photon correlation spectroscopy (PCS) (ZetaSizer, Malvern, Worcestershire, UK). For this, film fragments (0.1 g) were dispersed in 50 mL of ultrapure water (500× dilution). The nanostructures were extracted from the films under magnetic stirring for 2 h before analysis. For moisture assessment, the films were cut into 2 cm × 2 cm fragments and later placed in an oven at 60 °C [26]. These fragments were weighed after regular time intervals until the weight became constant. The residual water content in the films was determined following Equation (1).

Moisture content = [(Wd − Wi)/Wi] × 100(1)

where: Wd is the weight of the films after drying and Wi is the initial weight of the films.

To evaluate the swelling index, the films were cut into 2 cm × 2 cm pieces and weighed (Wd). Then, these fragments were placed in beakers containing 50 mL of pH 7.4 phosphate buffer at 37 °C for 24 h [26]. Afterwards, the films were removed from contact with the buffer and dried with absorbent paper, and the hydrated fragment was weighed (Ws). The swelling index was calculated following Equation (2).

Swelling index = [(Ws − Wd)/Wd] × 100(2)

where: Ws is the weight of the film after swelling and Wd is the weight of the dried film.

#### 2.2.4. Folding Endurance and Mechanical Properties

Folding endurance was determined by repeatedly folding the films in the same place up to 300 times (*n* = 3). Then, the films were evaluated for groove formation or breakage. The mechanical properties in terms of tensile strength, deformation and Young’s modulus was determined using the universal testing machine (Emic, São José dos Pinhais, Brazil), according to ASTM-D882-02 standards [29]. For this, film samples measuring 60 mm × 45 mm and with about 40 µm thick were individually fixed on the machine probe and a tensile load was applied at an initial separation of 4 cm and 50 mm/min of the cross-head speed. The maximum deformation suffered by the film was determined by the percentage change in the length of the sample in relation to its original size. The tensile strength was determined by the ratio of the force needed to rupture the film and the cross-sectional area of the strip, whereas the young’s modulus was calculated by the ratio of stress and strain values.

#### 2.2.5. In vitro Studies

##### Occlusion Test

The in vitro occlusion test was carried out according to our previous protocol [27]. For this, a 100 mL capacity beaker containing 50 mL of water was sealed with a cellulose acetate filter (90 mm, Sterlitech, Auburn, USA), which was subsequently covered with the bilayer films (*n* = 3). The films were applied with the pullulan layer in contact with the filter paper. Then, the beakers were stored at 32 °C and at predetermined times (0, 6, 24 and 48 h) they were weighed for the water loss determination. A film-free beaker was used as a negative control and the occlusion factor was calculated according to Equation (3).

Occlusion factor = [(A − B)/A] × 100(3)

where: A is the water loss of the negative control and B is the water loss in film presence.

##### Skin Preparation

Human skin fragments were obtained from healthy female patients undergoing abdominoplasty surgery. Subcutaneous fat was removed and the skin was stored at −4 °C (freezer) until use. Two different skin conditions were obtained. The first condition was the whole skin (intact cutaneous tissue), with the presence of *stratum corneum*, epidermis and dermis. The second condition was skin with an impaired barrier in which the *stratum corneum* was removed by a successive tape-stripping procedure [30] using 18 pieces of adhesive tape. The protocol was approved by the research committee with humans from the Federal University of Santa Maria—RS without identifying data (CAEE: 27168719.4.0000.5346).

##### Bioadhesive Strength

To carry out the experiment, an adapted apparatus was used composed of two balanced arms [31]. A plastic frame was connected to one of these arms under which the films were fixed. The skin (intact or injured) was fixed on a glass plate under the frame. The contact between the films and the skin fragment occurred by applying a weight of 1 N for 60 s. Afterwards, water was added at a constant speed of 1 drop/s in an opposite side plastic tube until the separation between skin and film occurred.

All analyzes were performed in triplicate and the volume of water used was measured in a graduated cylinder. Both the top layer (gellan gum layer) and the bottom layer (pullulan layer) of the bilayer films were analyzed. The bioadhesive strength was calculated using Equation (4) and the result was expressed in dyne/cm^2^.

Bioadhesive strength (dyne∕cm^2^) = (V × G)/A (4)

where: V = amount of water (g) required for the detachment between the sample and the tissue; G = acceleration of gravity (980 cm/s^2^); A = area of exposed tissue (cm^2^).

##### SB Release and Skin Permeation/Penetration Study

The in vitro SB release and skin permeation/penetration from films was conducted through vertical Franz diffusion cells with diffusion area of 3.14 cm^2^. The receptor medium used in the assays was pH 7.4 phosphate buffer at 32 ± 0.5 °C. The films (1 cm × 1 cm) corresponding to 440 μg of SB were placed on a dialysis membrane (10,000 Da, Sigma Aldrich, São Paulo, SP, Brazil) or on the skin surface with the pullulan layer in contact with the donator medium.

For the release study, at predetermined periods (1, 2, 3, 4, 5, 6, 7, 8, 12 and 24 h), a volume of 300 μL of the receptor medium was removed and replaced by the same volume of medium fresh. The amount of SB released was determined using HPLC method described in the Section 2.2.3. For the skin permeation study, skin samples were obtained and treated as described in the Section ”Skin Preparation”. This experiment was performed using intact (separating the skin layers only at the end of 24 h) and injured skin (without *stratum corneum*). The circular skin fragments were placed between the donor and recipient compartments with the dermis in contact with the recipient medium. After 24 h, the films were gently removed from the skin surface, the skin was carefully removed and the receptor medium was collected. For intact skin, the *stratum corneum* was removed using 18 pieces of adhesive tape. For both intact and injured skin, the epidermis was separated from the dermis by heating the skin in ultrapure water at 60 °C for 45 s, followed by removing the epidermis with a spatula. The strips containing the *stratum corneum*, and the epidermis and dermis fragments were placed in different test tubes containing methanol and vortexed for 2 min followed by an ultrasound bath for another 30 min. The SB content in the different skin layers and in the receptor medium was determined by HPLC (Section 2.2.3).

##### Free Radical Scavenging Activity

The antioxidant effect of the film containing nanoencapsulated SB was evaluated through the ability to scavenge the synthetic radical 2,2’-azinobis (3-ethylbenzothiazoline-6-sulfonic acid) (ABTS^+^), as previously described by Yang et al. [32], with some modifications. First, an ABTS^+^ solution was prepared by reacting the ABTS stock solution (7 mM) with sodium persulfate (140 mM), 12 h before the assay (final ABTS^+^ concentration 42.7 Μm). Bilayer films were cut into 0.5 cm × 0.5 cm pieces and added to tubes containing 1 mL of ABTS^+^ solution. The tubes were mixed by inversion and incubated at room temperature for 30 min. An ABTS^+^ solution was kept under the same reaction conditions and was used as a negative control. Blank samples containing the film fragments and water were also prepared. After incubation, the films were removed and the absorbance of solution was measured at 734 nm (UV-1800 Spectrophotometer, Shimadzu, Kyoto, Japan). The experiment was carried out in triplicate. Percentage radical scavenging capacity was calculated using the Equation (5).

SC% = 100 ((AbsA − AbsB) × 100)/AbsC(5)

where: SC%: Scavenging capacity in percentage; AbsA: sample absorbance; AbsB: blank absorbance; AbsC: negative control absorbance.

##### Hemocompatibility Evaluation of Formulations

The films hemocompatibility was evaluated by direct contact test according to Standard Practice for Assessment of Hemolytic Properties of Materials [33], with some modifications. For this purpose, anti-coagulated blood (9 parts of blood to 1 part of citrate) was collected from a healthy human volunteer. Then, 2 mL of the anticoagulated blood was centrifuged at 2000 rpm for 5 min, followed by discarding the plasma. The resulting pellet was washed with saline solution 3 times to completely remove plasma and obtain only erythrocytes. Afterwards, the erythrocytes were resuspended in saline and at a final concentration of 10% (*v*/*v*). Film fragments measuring 0.5 cm × 0.5 cm were inserted into microtubes containing 700 μL of saline solution and allowed to equilibrate for about 1 h. Afterwards, 100 μL of resuspended erythrocytes were added to the tubes. Positive and negative hemolysis controls were prepared with water and saline, respectively. In addition, blanks were prepared containing the film fragments and water. The tubes were then incubated for 1 h at 37 °C. After incubation, all samples were centrifuged at 2000 rpm for 5 min and the absorbance of the supernatant was measured spectrophotometrically at 540 nm (UV-1800 Spectrophotometer, Shimadzu, Kyoto, Japan). The percentage of hemolysis was calculated according to equation 6. This protocol was approved by research committee with humans from the Federal University of Santa Maria—RS (CAEE: 27168719.4.0000.5346).

% of hemolysis = (AbA − AbB/AbC) × 100(6)

where: AbA: sample absorbance; AbB: blank absorbance; AbC: positive control absorbance.

#### 2.2.6. In Vivo Study

##### Animals

Female BALB-c mice (6–8 weeks old) were housed in a separate animal room at controlled temperature (22 ± 2 °C), under a 12/12-h light/dark cycle (the lights were turned on at 07:00 AM), with free access to standard diet and water. The experimental study was conducted according to the Ethical Research Committee of the Federal University of Pelotas, Rio Grande do Sul, Brazil and registered under the number CEEA 23357-2018/ 40-2019. The number of animals used was the minimum necessary to evaluate the consistent effects of the treatments and every effort was made to minimize their suffering.

##### Experimental Design

The experimental design of this study is illustrated in supplementary material (Figure S1). The allergen sensitization and challenge induced by DNCB lead to the development of skin lesions similar to those of AD, as previously described by Chan et al. [34]. The dorsal skin of each mouse was shaved to remove all hair from the area. In the sensitization phase, it was applied 200 µL of 0.5 *v*/*v*-% DNCB dissolved in acetone/olive oil (3:1 ratio) on the shaved area in the first three days of the experimental protocol. These animals were also challenged by applying 20 µL and 200 µL of 1.0 *v*/*v*-% DNCB on the right ear and the dorsal skin, respectively, on days 14, 17, 20, 23, 26 and 29 of the experimental protocol.

In order to evaluate the effects of free SB or bilayer films treatments on the AD-like skin lesions, mice were randomly divided into seven experimental groups (*n* = 7 animals/group): normal control mice were exposed to the vehicle containing acetone/olive oil (3:1) and AD-induced mice were sensitized and challenged with DNCB. All other experimental groups were sensitized and challenged with DNCB, as well as received the following treatments: the Free SB (500 µL); the bilayer vehicle film (BF vehicle) (2.5 H × 2.5 L); the bilayer film containing NC without SB (BF NC B) (2.5 H × 2.5 L), the bilayer film containing nanoencapsulated SB (BF NC SB) (2.5 H × 2.5 L) or 1% of hydrocortisone (HC) (0.5 g), as a comparative drug commonly prescribed for the AD treatment. The Free SB solution was prepared by dissolving the SB in 10 mL of acetone/olive oil (3:1).

The treatments mentioned above were applied in the dorsal region of mice and secured with a bandage starting on day 14 of the experimental protocol. At the same days of mice were challenged with DNCB (14, 17, 20, 23, 26 and 29), the films were changed. The animals were monitored in order to ensure that the films were not removed from the application site. The treatment schedule was based on previous studies that used the same animal model and assessed the pharmacological action of films formulations [35,36].

Followed the last treatment, on day 30 of the experimental protocol, the scratching behavior, one of the hallmark AD-like behaviors, was evaluated in the animals. Twenty-four hours later (day 31), the clinical skin severity scores were determined to assess the manifestations of the AD-like signs in mice. After that, the animals were euthanized by inhalation of isoflurane anesthetic. The samples of dorsal skin of each mouse were rapidly dissected, weighted and frozen at −20 °C to further biochemical analyses. In addition, both ears and the spleen were collected to determine the ear edema and the spleen index, respectively.

#### 2.2.7. AD-like Clinical Signs

##### Scratching Behavior

The scratching behavior, one of the classic AD-like signs, was evaluated on day 30 of the experimental protocol. The time that mice spent rubbing the dorsal skin, ears and nose with their hind paws was measured and recorded for 30 min [37]. The data was expressed in seconds (s).

##### Clinical Skin Severity Scores

On day 31 of the experimental protocol, the dorsal skin of mice was photographed and the skin severity scores were assessed according to the method described by Park and Oh [38]. The characteristic signs of skin lesions were classified as: (1) pruritus/itching, (2) erythema/hemorrhage, (3) edema, (4) excoriation/erosion and (5) scaling/dryness. The above-mentioned signs were ranked as: 0 (no signs), 1 (mild), 2 (moderate) and 3 (severe).

#### 2.2.8. Evaluation of the Inflammation Markers

##### Ear Swelling

On day 31 of the experimental protocol, the animals were euthanized and both ears were cut at the base and weighted on the analytical balance. The ear swelling was measured by the difference between the samples of the DNCB-treated ear (right) and the control ear (left). The results were expressed in mg.

##### NOx Content

The samples of dorsal skin were homogenized in ZnSO_4_ (200 mM) and acetonitrile (96%). The homogenates were then centrifuged at 14,000 rpm for 30 min at 4 °C, and the supernatant was collected for the NOx assay. The accumulation of nitrite in the supernatant, an indicator of NO oxidation, was assessed by Griess reaction [39]. Briefly, the NOx content was estimated in a medium containing 2% vanadium chloride (in 5% HCl), 0.1% N-(1-naphthyl) ethylenediamine dihydrochloride and 2% sulfanilamide (in 5% HCl). After incubation at 37 °C for 1h, the color reaction was measured spectrophotometrically at λ = 540 nm. The concentration of nitrite/nitrate in the supernatant was determined from a sodium nitrite standard curve and expressed as nmol NOx/g of tissue.

#### 2.2.9. Evaluation of the Immune Function

##### Spleen Index

On day 31, mice were sacrificed and the spleen were harvested and weighted on the analytical balance to calculate its relative weight trough the formula: Spleen (g)/Body weight (g). The results were expressed as spleen index.

#### 2.2.10. Evaluation of the Oxidative Stress Markers

##### Tissue Preparation

To elucidate the involvement of oxidative stress, the samples of dorsal skin were homogenized (1:10, *w*/*v*) in 50 mM Tris-HCl at pH 7.4. The homogenates were centrifuged at 2500 rpm for 10 min and the low-speed supernatant (S1) was used to determine the thiobarbituric acid reactive species (TBARS) and non-protein thiol (NPSH) levels as well as the catalase (CAT) activity.

##### Protein Concentration

The protein concentration was estimated according to the method described by Bradford [40], using a bovine serum albumin (1 mg/mL) as a standard. The color reaction was measured spectrophotometrically at λ = 595 nm.

##### TBARS Levels

TBARS assay was performed to indirectly determine the malondialdehyde (MDA) levels, an important lipid peroxidation marker. As previously described by OHKAWA et al. [41], MDA reacts with 2-thiobarbituric acid (TBA) under acidic conditions and high temperatures to yield the chromogen. The S1 aliquots were incubated with 0.8% TBA, acetic acid buffer (pH 3.4) and 8.1% sodium dodecyl sulfate (SDS) for 2 h at 95 °C. The color reaction was measured at λ = 532 nm and the results were expressed as nmol of MDA/mg of protein, respectively.

##### NPSH Levels

The NPSH, a non-enzymatic antioxidant defense, was determined by Ellman’s method [42]. Briefly, S1 was mixed (1:1) with 10% trichloroacetic acid. After centrifugation (3000 rpm for 10 min), an aliquot of supernatant containing free SH-groups was added in 1 M potassium phosphate buffer pH 7.4 and 10 mM 5,5′-dithiobis-2-nitrobenzoic acid (DTNB). The color reaction was measured at λ = 412 nm and NPSH levels were expressed as nmol of NPSH/g tissue.

##### CAT Activity

CAT activity was spectrophotometrically determined by monitoring the H_2_O_2_ consumption at λ = 240 nm, as previously described [43]. In a medium containing an aliquot of S1 and 50 mM potassium phosphate buffer pH 7.0, the enzymatic reaction was started by adding the substrate H_2_O_2_ (0.3 mM). The enzymatic activity was expressed as Units (1U decomposes 1 mmol of H_2_O_2_ per minute at pH 7.0 and 25 °C)/mg protein.

#### 2.2.11. Statistical Analysis

Formulations were prepared and analyzed in triplicate and the results were expressed as mean ± standard deviation (SD) or standard error of the mean (SEM). A Gaussian distribution was tested using D’Agostino normality test. For data considered parametric, an one or two-way analysis of variance (ANOVA) followed by post-hoc Tukey’s test was performed to compare the significant difference among the experimental groups. All statistical analyses were performed using GraphPad Prism statistical software (version 8.0, San Diego, CA, USA. Values of *p* < 0.05 were considered statistically significant.

## 3. Results

### 3.1. The Bilayer Films Presented Suitable Physicochemical Characteristics

**Figure 1:**
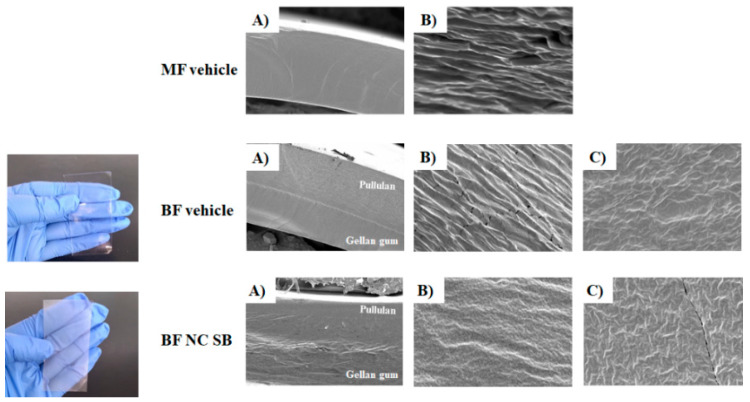
Macroscopic appearance and comparative SEM images of the side sections of monolayer vehicle film (MF vehicle), bilayer vehicle film (BF vehicle) and bilayer film containing silibinin-loaded nanocapsules (BF NC SB) with magnification of 300× (**A**) and magnification of 5000× on the gellan layer (**B**) and pullulan layer (**C**).

Firstly, NC suspensions were produced and characterized. These NC presented particle diameter of 115 ± 3 and 134 ± 5 nm for SB-loaded NC and unloaded NC, respectively. The polydispersity index was below 0.2 and the zeta potential was around −10 mV for both NC suspensions. Besides, the content of SB was 98.9%, being suitable for incorporation into the films. Then, bilayer films were prepared using gellan gum as the first polymeric layer forming agent and pullulan as the second layer. The prepared films presented a macroscopically homogeneous appearance. Besides, the BF NC SB was slightly whitish due the nanostructure presence (Figure 1). Figure 1A shows the images obtained from the side sections of the films at a magnification of 300×. The bilayer formation was clearly observed, without visualization of detachment between the different layers, confirming their adhesion. Besides, the interface between the layers of vehicle film was slightly delimited, which was probably due to the high affinity existing between the gellan gum and pullulan. Whereas, for the films containing the NC, it was observed an irregular interface, probably due to the partial nanoparticles migration from the gellan gum layer to the pullulan layer during the process of the second layer formation. However, it was not possible to observe the presence of these nanostructures in the different polymeric layers at higher magnification (Figure 1B,C), indicating that the nanostructures are intimately embedded between the chains of the film-forming polymers.

Then, bilayer films were examined for thickness, moisture, content homogeneity, swelling index and mechanical properties (Table 1). Analysis of the homogeneity of dosage and thickness were performed to ensure the consistency of dosage and of film formation along the extended area. We found that the films had a homogeneous thickness with values close to 40 µm, as well as the film fragments presented around 440 µg/cm^2^ of SB, which represents about 92.25% of theoretical value (477 µg/cm^2^).

After NC incorporation into bilayer film, it was observed that their nanometric size was maintained. In addition, this inclusion reduced the swelling index and deformation values of the film, as well as increased the tensile strength and Young’s Modulus values (*p* < 0.05). The folding endurance test was manually measured in which no film showed formation of cracks or breaks after being folded in the same place for 300 times, suggesting adequate flexibility.

### 3.2. The Nanocapsules Improved the Occlusive Potential of Bilayer Film

The result of the occlusion test is presented in Table 2. The occlusion factor of nano-based films was significantly higher than BF vehicle at all analyzed time points (*p* < 0.001), indicating that the incorporation of NC into the polymeric matrix increased the capacity of water retention by the film. Besides, no significant difference (*p* > 0.05) was observed between films containing unloaded and the corresponding SB-loaded NC, suggesting that flavonoid encapsulation did not change the occlusion factor.

### 3.3. The Pullulan Layer Confers Bioadhesion to the Film

**Figure 2:**
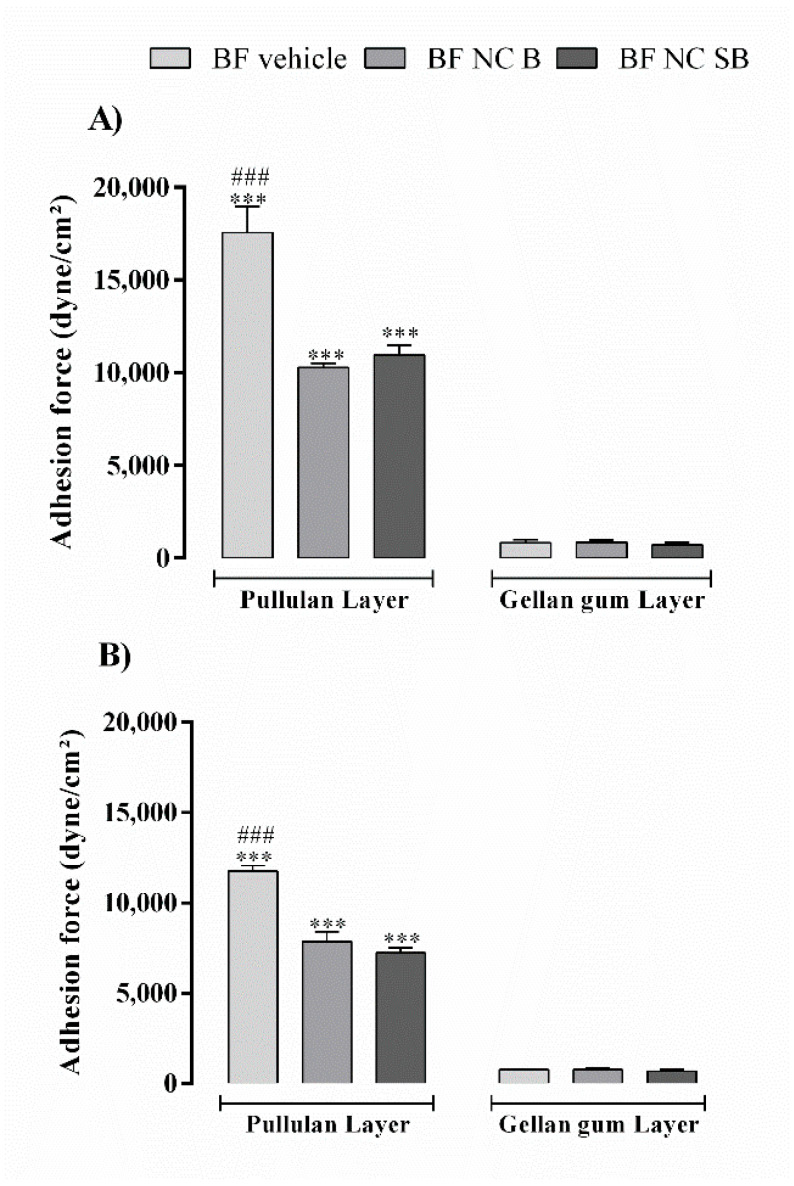
In vitro bioadhesion evaluation using the non-injured (**A**) and injured (**B**) skin of bilayer vehicle film (BF vehicle) and films containing nanocapsules with silibinin (BF NC SB) or without (BF NC B). Each column represents the mean ± SD. The films were evaluated on both sides (pullulan layer and gellan gum layer). (***) *p* < 0.001 significant differences between pullulan layer and gellan gum layer, (###) *p* < 0.001 significant differences between BF vehicle and BF NC SB or BF NC B (Two-way ANOVA followed by Tukey’s test).

Figure 2 shows the results obtained after evaluating the bioadhesive strength of bilayer films using two skin conditions: full thickness and superficial lesion. In both skin conditions evaluated, the films showed higher values of bioadhesive strength when evaluated with the pullulan layer in contact with the skin surface (*p* < 0.001). Bioadhesive strength values were reduced after the NC incorporation into films (*p* < 0.001). The skin condition studied also influenced the films bioadhesion, presenting a reduction in the values of 17,554 ± 1399 to 11,753 ± 528 dyne/cm^2^ and of 10934 ± 699 to 7230 ± 863 dyne/cm^2^ for BF vehicle and BF NC SB, respectively.

### 3.4. The BF NC SB Slowly Releases SB and Retains it in the Skin

**Figure 3:**
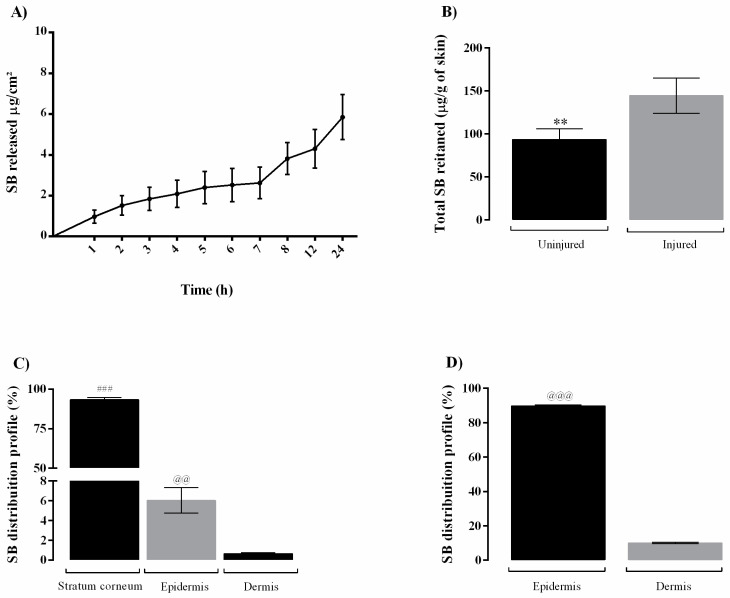
In vitro release profile and skin permeation of silibinin from nano-based bilayer film. (**A**) Cumulative amount of released silibinin from film in phosphate buffer receptor medium pH 7.4 at 32.0 °C (*n* = 3); (**B**) Cumulative amount of retained silibinin in the uninjured and injured skin after 24 h of incubation (*n* = 6); (**C**) Percentage of silibinin distribution in the different non-injured skin layers; (**D**) Percentage of silibinin distribution in the different injured skin layers. All the results are expressed as mean ± SEM. (**) *p* < 0.01 significant differences between cumulative amount of SB in the non-injured and injured skin, (###) *p* < 0.001 significant differences between SB quantified in the *stratum corneum* and epidermis in the non-injured skin, (@@) *p* < 0.01 and (@@@) *p* < 0.001 significant differences between compound quantified in the epidermis and dermis in the non-injured or injured skin.

The SB release from the films is represented by the cumulative release of substance per area as a function of time in hours (Figure 3A). The BF NC SB film presented a slow release over the period of 24 h, reaching 7.14 ± 1.34% of SB released.

In relation to the skin permeation of SB from the bilayer film, the total SB retained (Figure 3B) on the uninjured was less than injured skin (*p* < 0.01). Besides, regardless the skin condition (uninjured and injured) the SB was not detected in the receptor medium. The Figure 3C,D show the distribution percentage of SB in different layers of skin after 24 h of experiment for uninjured and injured human skin, respectively. In both skin conditions, SB penetrated the cutaneous tissue in quantifiable drug amounts. In uninjured skin, the flavonoid accumulated preferably in the *stratum corneum*. For the injured skin a higher SB amount in the epidermis in comparison to dermis was observed (*p* < 0.001).

### 3.5. The BF NC SB Neutralized the ABTS+ Radical

**Figure 4:**
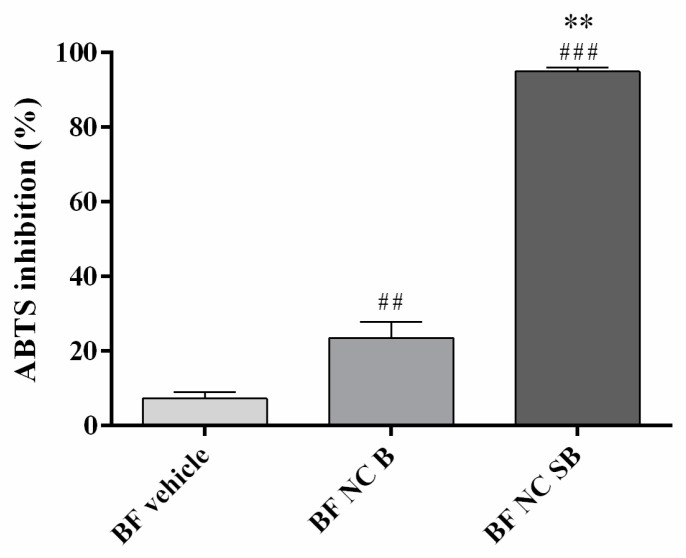
Percentage of ABTS radical inhibition by films. Each column represents the mean ± SD. (**) *p* < 0.01 significant differences between bilayer film containing nanocapsules with (BF NC SB) or without (BF NC B) silibinin, (##) *p* < 0.01 and (###) *p* < 0.001 significant differences between BF NC SB or BF NC B and BF vehicle (One-way ANOVA followed by the Tukey’s test).

Figure 4 shows radical scavenging activity of SB-loaded NC bilayer film in comparison to vehicle and bilayer film containing unloaded NC. Corroborating the antioxidant activity of SB, the BF NC SB presented high radical scavenging activity (about 100%). Both BF vehicle and BF NC B had a low influence on the neutralization of the ABTS+ radical, demonstrating that there is no false positive in the test performed.

### 3.6. The Bilayer Films Are Hemocompatible

The bilayer films were evaluated for their potential to cause lysis in human erythrocytes. The percentage of hemolysis was 0.73 ± 0.05%, 0.56 ± 0.23% and 0.61 ± 0.11% for vehicle film, film containing unloaded NC and film containing SB-loaded NC, respectively. All these values are similar to the hemolytic percentage of saline solution (0.70 ± 0.09%), indicating a good blood compatibility of produced films.

### 3.7. The BF NC SB Treatment Attenuated the AD-like Clinical Signs Induced by DNCB in Mice

**Figure 5:**
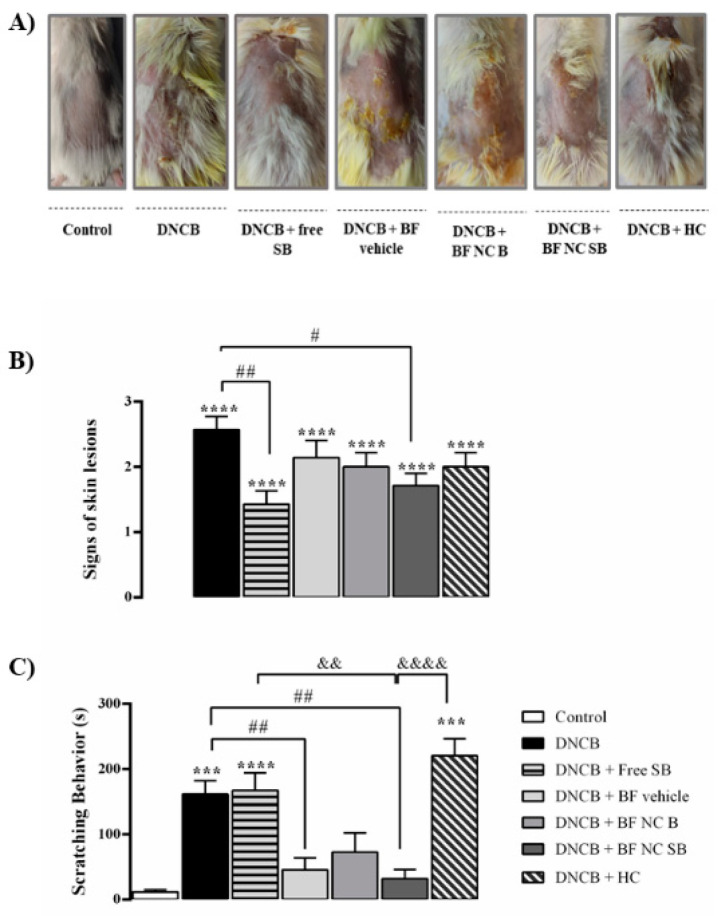
Effect of the different treatments on AD-like clinical signs induced by DNCB in mice. (**A**) Images of the skin lesions from the groups of mice taken on the last day of the experiment (day 31). (**B**) Score of the skin lesions. (**C**) Scratching time evaluated on day 30 of the experimental protocol. Each column represents the mean ± SEM of 7 mice per group. (****) *p* < 0.0001 and (***) *p* < 0.001 compared with the control group, (##) *p* < 0.01 and (#) *p* < 0.05 compared with the DNCB group, (&&&&) *p* < 0.0001 and (&&) *p* < 0.01 compared with the BF NC SB group (One-way ANOVA followed by the Tukey’s test).

The Figure 5 depicts the effect of free SB or BF NC SB treatments on the severity of the skin lesions and the scratching behavior in mice. Our data demonstrated that all the animals exposed to DNCB exhibited AD-like clinical signs, represented by an increase in the clinical skin severity score when compared with the control group (*p* < 0.0001). The free SB or the BF NC SB treatments markedly decreased the characteristics AD-like signs in the DNCB-exposed mice. In turn, the BF vehicle, the BF NC B and the HC did not alter the severity of lesions induced by DNCB.

The results demonstrated that the DNCB-exposed mice exhibited an increase in the scratching time when compared with the control group (*p* < 0.001). In contrast, both free SB and HC treatments did not alter the scratching behavior induced by DNCB in mice (*p* > 0.05). The treatment with BF NC B did not show statistical difference in the scratching time when compared to the DNCB- or vehicle-treated mice (*p* > 0.05). However, repeated appli-cations of the BF vehicle or the BF NC SB reduced this typical AD-like behavior in the DNCB group (*p* < 0.01). In fact, there is a statistically significant difference among the BF NC SB, HC, and free SB treatments, suggesting that the topical application of BF NC SB was more effective to reduce the scratching behavior in DNCB treated mice than the free SB (*p* < 0.01) or HC (*p* < 0.0001).

### 3.8. The BF NC SB Treatment Suppressed the Ear Swelling Induced by DNCB in Mice

**Figure 6:**
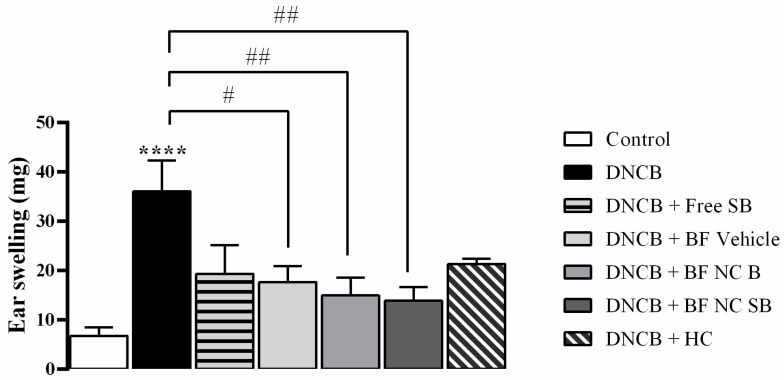
Effect of the different treatments on the ear swelling induced by DNCB in mice. The ear swelling was assessed on day 31 of the experimental protocol. Each column represents the mean ± SEM of 7 mice per group. (****) *p* < 0.0001 compared with the control group, (##) *p* < 0.01 and (#) *p* < 0.05 compared with the DNCB group (One-way ANOVA followed by the Tukey’s test).

The Figure 6 illustrates the effect of free SB as well as all formulations containing or not SB on the development of ear swelling induced by DNCB in mice. The results evidenced that the DNCB substantially increased the ear swelling when compared with the control group (*p* < 0.0001), whereas the topical application of BF vehicle, BF NC B and BF NC SB reduced the ear swelling induced by DNCB in mice (*p* < 0.01). On the other side, the treatments with free SB or HC had no statistical difference on ear swelling when compared to vehicle or DNCB exposed mice (*p* > 0.05).

### 3.9. The BF NC SB Treatment Did Not Alter the Spleen Index after DNCB Exposure in Mice

**Figure 7:**
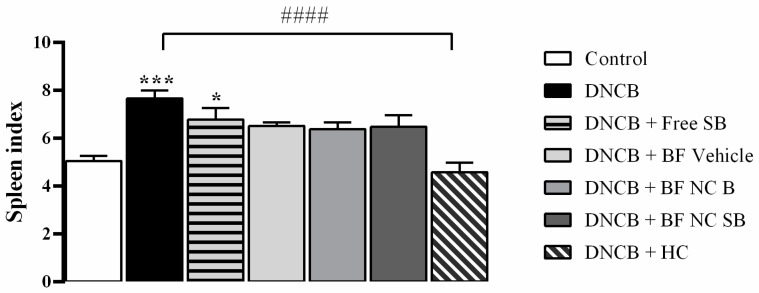
Effect of the different treatments on the spleen index. This parameter was assessed on day 31 of the experimental protocol. Each column represents the mean ± SEM of 7 mice per group. (***) *p* < 0.001 and (*) *p* < 0.05 compared with the control group, (####) *p* < 0.0001 compared with the DNCB group (One-way ANOVA followed by the Tukey’s test).

As shown in Figure 7, the DNCB-exposed mice significantly increased the spleen index in comparison with the control group (*p* < 0.001). Only the HC treatment attenuated the splenomegaly induced by DNCB in mice (*p* < 0.0001). No statistically significant difference was evidenced in the spleen index after the topical applications of BF vehicle, the BF NC B, and the BF NC SB in the dorsal skin of mice when compared to DNCB or control groups (*p* > 0.05)

### 3.10. The BF NC SB Treatment Modulated Some Markers of Oxidative Stress and Inflammation in the Dorsal Skin of Mice Exposed to DNCB

**Figure 8:**
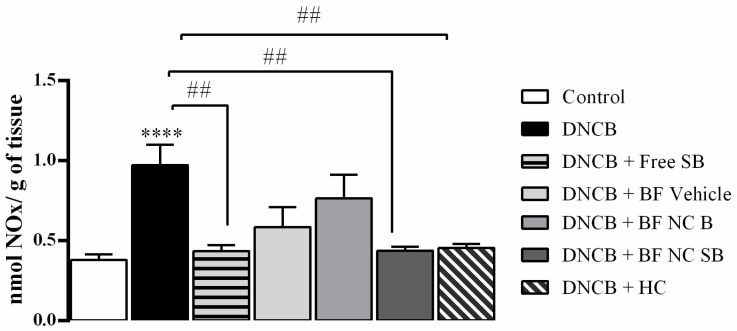
Effect of the different treatments on the NOx levels in the dorsal skin of mice exposed to DNCB. Each column represents the mean ± SEM of 7 mice per group. (****) *p* < 0.0001 compared with the control group and (##) *p* <0.01 compared with the DNCB group (One-way ANOVA followed by the Tukey’s test).

The dorsal skin of DNCB-treated mice exhibited a significant excessive production of NOx levels when compared with the control group (*p* < 0.0001), as shown in Figure 8. The topical applications of free SB, BF NC SB and HC reduced the NOx levels in the dorsal skin of DNCB exposed mice (*p* < 0.01) whereas the treatments with BF vehicle or BF NC B had no statistical difference on the NOx levels in the dorsal skin of mice when compared to vehicle or DNCB groups (*p* > 0.05).

**Figure 9:**
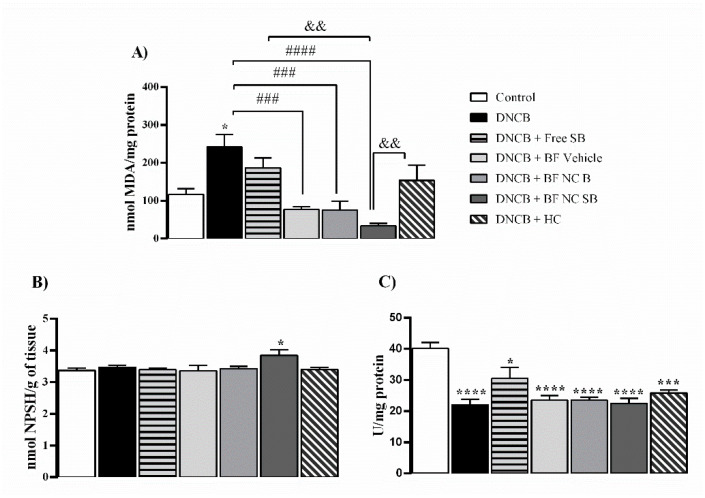
Effect of the different treatments on the levels of TBARS (**A**) and of NPSH (**B**) as well as on the CAT activity (**C**) in the dorsal skin of mice exposed to DNCB. Each column represents the mean ± SEM of 7 mice per group. (****) *p* < 0.0001, (***) *p* < 0.001 and (*) *p* < 0.05 compared with the control group, (####) *p* < 0.001 and (###) *p* < 0.01 compared with the DNCB group, (&&) *p* < 0.01 compared with the BF NC SB group (One-way ANOVA followed by the Tukey’s test).

The Figure 9 summarizes the results regarding the effect of the film formulations in some oxidative stress parameters in the dorsal skin of mice exposed to DNCB. In relation to the control group, the DNCB exposure led to an enhancement of TBARS levels in the dorsal skin of mice (*p* < 0.05). In turn, the treatments with BF vehicle, BF NC B, as well as BF NC SB reduced the TBARS levels in animals exposed to DNCB (*p* < 0.0001). The topical applications of free SB or HC did not alter the TBARS levels in the dorsal skin when compared to vehicle or DNCB groups (*p* > 0.05). In this line, the BF NC SB treatment was statistically more effective in reducing the TBARS levels in the dorsal skin of mice exposed to DNCB than the free SB or HC (*p* < 0.01) (Figure 9A).

Regarding the non-enzymatic antioxidant defenses, the repeated applications of BF NC SB increased the levels of NPSH in the dorsal skin of mice in relation to the control group (*p* < 0.05) whereas the statistical analysis also revealed similar NPSH levels in the dorsal skin of mice among all other experimental groups (*p* > 0.05) (Figure 9B). Moreover, the animals exposed to DNCB presented an inhibition of CAT activity in the dorsal skin when compared with the control group (*p* < 0.0001). All the formulations tested, as well as the positive control (HC) were unable to restore the CAT activity at the control levels in the dorsal skin of DNCB-treated mice (*p* > 0.05) (Figure 9C).

## 4. Discussion

Aiming to suppress the clinical AD symptoms, this study was designed such that the immediate layer composed by pullulan could provide an adhesion to the cutaneous tissue, whilst a gellan gum layer containing SB-loaded NC provides a therapeutic effect in sustained manner. For this, firstly, the bilayer films were produced by applying a second pullulan layer on top of the first layer composed by gellan gum incorporated with SB-loaded NC using two-step solvent casting method. Micrographs of side sections of produced films exhibited clearly the formation of two polymeric layers, which seem to be adhered to each other, without gap signals.

After drying, is important to assure the homogeneity of the final formulation, since during this process may be observed agglomeration or sedimentation of solid particles or air bubbles on the surface, leading to homogeneity problems [19]. The bilayer films produced presented a thin and homogeneous thickness, which is required for cutaneous application. In addition, the SB content was in the range of 85 to 115% recommended for polymeric films [44], as well as this flavonoid was evenly distributed throughout the film as demonstrated by the content uniformity test. The residual water content in the films was less than 15% for all films, corroborating the previous reports for films produced by the same technique [26,27]. All these evaluations indicate that the casting solvent method employed to produce the bilayer films was successful.

In the SEM analysis, although a difference was observed between the microstructure of vehicle film and of nano-based film, it was not possible to visualize the NC. However, PCS analysis indicated the nanometric size maintenance of particles in the final dosage form, suggesting that the NC are intimately inserted between the polymeric chains. The nanoparticles presence in the polymeric film network has already been suggested in others researches involving polymeric films containing nanostructured systems [18,45]. This insertion may restrict the free movement of polymer chains [46]. In fact, our results demonstrated that the films containing NC showed higher young’s modulus values and lower swelling index than vehicle film, indicating a reduction of both mobility and relaxation of the polymeric chains, making the film stiffer and less susceptible to penetration by fluids. However, all the produced bilayer films showed fluid absorption capacity, as well as remained intact after 24 h in contact with the buffer, suggesting that they are a resistant material for the exudative lesions treatment. This result may be due to the presence of the gellan gum layer in the film, which forms swellable and resistant films, being replaced less frequently in the lesion site [20,21]. Moreover, the young’s modulus obtained for the developed films have values within of the young’s modulus range of skin, which can vary between 0.02 and 57 MPa [47].

The formation of two distinct layers in the film was also confirmed through the bioadhesion test, in which the layer composed by pullulan presented values of bioadhesive strength greater than the layer composed by gellan gum. In fact, pullulan-based formulations have already been reported in the literature with bioadhesive properties [22,48]. The bioadhesion can contribute to an intimate contact of the pharmaceutical dosage form with the skin, increasing the residence time at the action site and decreasing the administration frequency. Besides, the use of bioadhesive films may reduce the use of adhesive tapes for their application, which are constantly associated with greater skin irritation and pain [49]. Considering that AD is characterized by a loss of skin barrier function, mainly due to a damage to the lipids of the *stratum corneum* [1], the bioadhesion was also evaluated using an injured skin model (without *stratum corneum*). The films bioadhesive potential was lower when in contact with the injured skin. Similar results were observed for hydrogels containing β-caryophyllene nanoemulsions [50]. Besides, the bioadhesion values of nano-based films were lower than vehicle film in both uninjured and injured skin conditions. In fact, it was observed an irregular interface in the SEM images for film containing NC. This result reinforces our argument that NC migrate from the gellan gum layer to the pullulan layer during the film formation.

Distinct theories are used to describe the bioadhesion. Among these theories is adsorption, in which the bioadhesiveness between a substance and a tissue results from van der Waals, hydrophobic or hydrogen bond interactions [51]. The hydrophilic groups of pullulan may be preferentially oriented towards the inside of the film interacting with glycerol used as plasticizers, as showed in others studies involving hydrophilic polymers-based films [49,52]. Thus, the film surface may become slightly more hydrophobic, favoring its interaction with lipophilic surface of *stratum corneum* in uninjured skin. However, simulating a skin damage condition through *stratum corneum* removal occurs viable epidermis exposure, which is less lipophilic than *stratum corneum*, reducing the pullulan interaction. This greater bioadhesion in the intact skin than in the injured skin can be beneficial, since during the film peeling from skin, the lesion area will not be more damaged. In addition, the NC inclusion in polymeric chains may be masking the chemical groups in pullulan that interact with the skin, resulting in lower bioadhesion. In fact, in others studies were also observed a bioadhesive reduction of hydrophilic polymers after inclusion of solid substances into films [49,53].

The skin barrier impairment in patients with AD leads to the transepidermal water loss, as well as the drug permeability barrier is diminished in these patients, increasing the risk of systemic drug absorption [1]. The in vivo skin hydration can be correlated with in vitro occlusion factor in a linear form. In other words, the greater the occlusion factor, the greater the cutaneous hydration observed in vivo [54]. Our results confirm the higher occlusive effect provided by nanostructures, as previously reported in other studies [18,27]. This result points out that nano-based films are promising in the design of novel treatments intended for improving skin hydration.

Regarding permeation study, in the intact skin, the BF NC SB providing a SB deposition in higher amounts in the *stratum corneum* followed by its delivery on epidermis and dermis. This find is in line with the cutaneous permeation mechanism observed for polymeric nanoparticles reported in the literature [55]. As expected, after removing the main barrier to substances penetration across the skin, an increase in the SB accumulation in the epidermis and dermis was observed. However, despite the SB amount increased in the injured skin, no amount of this flavonoid was detected in the receptor medium. This affinity for cutaneous tissue is advantageous for AD treatment because favors a site-specific therapeutic response, without systemic absorption [56]. In addition, in vitro release test evidenced a sustained SB release from the nano-based film. This controlled release may favor the SB delivery locally in the skin in a well-controlled manner, allowing a less frequent film replacement and avoiding greater local irritation and the risk of injured area contamination [26,56].

Previously published data showed that gellan gum films had young’s modulus about three times greater and occlusion factor about twice lower than bilayer films produced [27]. Besides, this same gellan gum film presented SB amount released from nano-based film around 5% in 24 h under the same conditions, as well as similar SB distribution profile in the different skin layers to that found for bilayer films. The improvement of young’s modulus and occlusive properties observed here are in line with described in the literature for bilayer films. This type of film presents heterogeneous structures, taking advantage of the best characteristics of each individual polymer, and thus, improving the physical and mechanical properties of the final film [24]. Besides, pullulan is highly hydrophilic, forming a fast-dissolving film [22]. Thus, it is possible to infer that the pullulan layer addition confers an increase in bioadhesion and occlusion values, without altering the release and skin permeation profile of SB.

Flavonoids have antioxidant and anti-inflammatory effects which may ameliorate signs of allergic diseases, including AD [4]. Besides, studies describe that the beneficial effects of SB in skin pathologies are mainly due to its action in preventing the generation of oxidative stress. Thus, a preliminary in vitro evaluation of the SB antioxidant effect from the nano-based bilayer films produced was performed. The results showed a high capacity of BF NC SB to neutralize the ABTS+ radical, corroborating others studies that evaluated the antioxidant effect of this flavonoid using this same synthetic radical [32,57]. Next, the hemocompatibility of the developed bilayer films was determined in order to assess their safety when in contact with red blood cells, since AD lesions may bleed. Our results showed a hemolytic index less than 1% for all the bilayer films. According to the Standard Practice for Assessment of Hemolytic Properties of Materials, materials that cause 0–2% hemolysis are considered non-hemolytic and safe [33].

Given the in vitro results obtained, it was performed a pre-clinical study to evidence the therapeutic efficacy of bilayer films against AD using hapten-induced mice. AD is characterized by itchy, red and swollen skin due to chronic inflammation and immune dysregulation. Multiple challenges with haptens, such as DNCB, into the skin of mice mimics the ongoing maintenance and the progression of AD. Thereby, the epicutaneous sensitizer DNCB is commonly used to induce a chemical animal model of dermatitis [58]. The present study reinforces that DNCB model reproduced the typical AD-like signs, as evidenced by an increase in the severity of skin lesions and the scratching behavior. Considered a typical AD symptom, scratching could be responsible for trigger a physical injury to epidermis, as well as aggravate the inflammatory processes. In this line, it is recognized as an essential and a skin specific behavior related to itching [59].

Immunologically, it has been reported that haptens exposure for an extend period evoke primarily the release of T helper 1 (Th1) cytokines that shift to a delayed chronic Th2-dominated inflammatory response, that it is similar to human AD. In this context, the immunological and inflammatory process is closely implicated in the pathogenesis of AD [60]. Some studies established that the local inflammation and the skin irritation induced by the external application of DNCB could also result in ear edema, probably due to an increase in the vascular permeability [36,37]. As expected, our results showed that repeated DNCB exposure promoted a marked ear swelling, as well as an enhancement in the spleen index and the NOx levels in the dorsal skin of mice. Interestingly, the spleen index, known as a peripheral immune organ, may be reflected the stimulation of immunological responses by activating T-lymphocytes [59]. Besides, it is well known that cytokines may lead to an excessive production of NO which aggravate the inflammatory response and consequently sustain the local tissue injury [61].

Previous studies have shown that melanocytes and keratinocytes, some types of skin cells, generates RS [62,63]. Similarly, the immune cells response and the release of inflammatory mediators are often associated with an increase in the production of oxidative molecules and reactive free radicals [64]. Indeed, an overproduction of NO levels lead to the development of oxidative stress that is also involved as a pathological aspect of AD [65,66]. In agreement with those findings, our results showed that repeated DNCB challenges inhibited the CAT activity and increased the TBARS levels, indicating the establishment of oxidative stress in the dorsal skin of mice. Indeed, these data reaffirm that the multifaceted interactions among the inflammatory mediators, the immune cells and the oxidative stress favor the maintenance of skin lesion like-AD.

Although topical corticosteroids are usually recommended as the standard anti-inflammatory treatment to alleviate the severe sings related to AD, their long-term use results in the manifestation of innumerous adverse effects, such as skin atrophy, purpura, dyspigmentation and declined immune function [67]. Consistent with these findings, our results revealed that mice treated with HC exhibited skin lesions like-AD, scratching behavior, a reduction in the spleen index and in the NOx levels on the local injury in mice. This data agrees with the fact that the long-term steroids use lead to the development of skin thinning, cracking or bleeding, as well as, immunosuppression [68].

All the bilayer films presented a significant reduction of scratching behavior, ear edema and TBARS levels whereas the other treatments did not reduce these parameters. It is known that the skin hydration is related to reduction of pruritus and skin barrier recovery, reducing the AD aggravation [69]. Consistent with this information, the in vitro results showed the bioadhesive and occlusive potential of films, suggesting that they may adhere to skin, improving it hydration. Similar result was obtained by Jeong and co-workers in which pullulan films with and without *Rhus verniciflua* extract ameliorated the AD-like sings as epidermal thickness and reduced the cell infiltration, suggesting that pullulan itself suppress AD development [16]. In fact, the pullulan layer was applied in contact with mice skin surface to act with bioadhesive function, while the gellan gum layer could act as external barrier against aggression and as vehicle for SB-loaded NC. In addition, both pullulan and gellan gum layers were plasticized with glycerol, a humectant used in dermatological formulations. Beyond to act as humectant, glycerol appears to accelerate the skin barrier repair after its disruption [9,69]. Thus, we speculate that bilayer films components may be promoting a physical barrier against scratches and other sensitizing agents, improving the hydration and restoring the skin’s barrier function.

Moreover, the current study demonstrated that free SB, BF NC SB and HC treatment reduced the NOx levels in the dorsal skin of mice after DNCB exposure. Considering the involvement of NO in inflammatory conditions, we speculated that a decrease in the NOx levels might trigger, at least in part, a decrease in vascular permeability as well as a lower production of cytokines and prostaglandins, thus decreasing the severity of the inflammatory process on the local injury [70]. NO, a highly reactive free radical, also contribute to oxidative damage that occur by lipid peroxidation and oxidation of proteins and thiols [66].

Although CAT activity was not altered by treatment with SB formulations, the BF NC SB treatment increased the levels of NPSH in the dorsal skin. Tripeptide glutathione (GSH), the major non-protein thiol quantified in the NPSH assay, plays an essential role in the cellular redox homeostasis, protecting organelles from oxidative damage and inflammatory cascade [71]. In this sense, our findings suggest that the BF NC SB enhanced the GSH production which led to an increase in the NPSH levels in an attempt to counteract the lipid peroxidation and the oxidative damage in the dorsal skin of DNCB exposed mice.

Our in vivo results confirmed the benefits of SB nanoencapsulation since topical applications of free SB alleviated the AD-like signs and NOx levels in mice whereas the BF NC SB decreased the incidence of the skin lesions score, the scratching behavior, the ear swelling and oxidative parameters. We believe that NC could increase the residence time of SB in the cutaneous tissue, and thus, exert a better antioxidant performance. In line with these findings, previous studies reported the promising anti-inflammatory effect of hydrogels containing nanoencapsulated SB in a model of contact dermatitis induced by croton oil [7] or DNCB [8]. In addition, specifically for AD, the better performance of polymeric nanoparticles in attenuating the inflammatory and immunological effects of the disease is well documented in the literature when compared to non-nanoencapsulated substances [56,72]. Recently our research group demonstrated that pullulan films incorporated with pomegranate seed oil NC presented better biological effects than this free vegetable oil or associated into nanoemulsions against the same mice model of AD used here, highlighting the superiority of NC formulations [18].

Collectively the results obtained in in vitro and in vivo evaluations suggest that bilayer films containing SB-loaded NC may exert a physical barrier action in AD treatment, as cutaneous bioadhesion, prevention of skin dryness, protection of lesions and ability to absorb exudates, followed by therapeutic action, with sustained and localized SB release and improved antioxidant effect. In this sense, the results obtained here encourage further investigations involving pullulan/gellan gum bilayer film as vehicle for AD control, as well as the combination of NC into films to improve the therapeutic performance. Furthermore, it is important to mention that, to our knowledge, this is the first study where a bilayer film based on gellan gum and pullulan containing nanostructures was produced, characterized and assessed against the AD injuries in a pre-clinical study.

## 5. Conclusions

This study demonstrated a facile prepare of gellan gum/pullulan bilayer, where the top pullulan layer was able to ensure a good bioadhesion to the skin while the bottom gellan gum layer has swelling capacity and acts as a vehicle for the release of SB-loaded NC. The in vitro studies showed that nano-based film presented higher occlusion factor. Besides, this film released SB in a slow and gradual manner and allowed the retention of this flavonoid in the skin tissue. Also, SB-loaded NC incorporated into bilayer films presented high scavenger capacity and did not present hemolytic degree. The in vivo study provided evidence that topical applications of BF NC SB attenuated the AD-like skin lesions, the scratching behavior and the ear edema by modulating some markers related to redox signaling and the inflammatory process. Interestingly, the bilayer film without SB presence attenuated the scratching behavior, the ear edema and TBARS levels, suggesting that the novel bilayer vehicle film might also provide benefits to treat atopic skin. It should be noted that the efficacy of BF NC SB treatment on behavioral and biochemical parameters was similar or better than the free SB solution, the classical treatment (HC) or bilayer films without SB. In this scenario, our data reinforce that the combination of NC and films could be applied to enhance the performance of antioxidant substances in skin disorders treatment. Particularly, we highlighted that the gellan gum/pullulan bilayer film containing SB-loaded NC might combine skin protection and hydration effects with improved anti-inflammatory and antioxidant effects, being a promising and potential therapeutic alternative for AD treatment.

## Figures and Tables

**Table 1:** Results of bilayer films characterization.

	BF NC SB	BF NC B	BF Vehicle
Drug content homogeneity (µg/cm^2^)	440.61 ± 5.21	-	-
Thickness (µm)	43 ± 7	42 ± 5	40 ± 5
Size (nm)	117 ± 11	137 ± 15	514 ± 143
Polydispersity index	0.28 ± 0.05	0.24 ± 0.05	0.47 ± 0.11
Swelling index (%)	106.95 ± 2.56 *	101.02 ± 6.45 *	134.42 ± 4.78
Moisture (%)	13.89 ± 2.01	12.83 ± 1.07	14.83 ± 2.64
Tensile strength (MPa)	1.09 ± 0.03 *	1.13 ± 0.09 *	0.51 ± 0.05
Elongation (%)	4.93 ± 0.45 *	4.61 ± 0.18 *	7.69 ± 0.15
Young’s modulus (MPa)	27.25± 5.95 *	24.25 ± 6.57 *	7.25 ± 5.95

The results are expressed by mean with SD of triplicate. Asterisks denote the significant difference (*) *p* < 0.05 by paired Student’s t test between BF vehicle and BF NC SB or BF NC B.

**Table 2:** Occlusion factor of bilayer films.

Formulation	Occlusion Factor (%)		
	6 h	24 h	48 h
BF Vehicle	20.18 ± 3.66 **	28.18 ± 2.05 **	29.45 ± 1.47 **
BF NC SB	51.90 ± 2.01	50.16 ± 1.84	55.78 ± 2.35
BF NC B	49.50 ± 4.31	52.30 ± 6.09	50.26 ± 2.45

The results are expressed by mean with SD of triplicate. Asterisks denote the significant difference by One-way ANOVA followed by the Tukey’s test. (**) *p* < 0.01 between BF vehicle and BF NC SB or BF NC B.

## Data Availability

Not applicable.

## Supplementary Materials

The following supporting information can be downloaded at: https://www.mdpi.com/article/10.3390/pharmaceutics14112352/s1, Figure S1: Schematic representation of the experimental design of in vivo study.
