# Supplementary material for: Novel Pullulan/Gellan Gum Bilayer Film as a Vehicle for Silibinin-Loaded Nanocapsules in the Topical Treatment of Atopic Dermatitis
Source: Pharmaceutics. 2022 Oct 31;14(11):2352. doi: 10.3390/pharmaceutics14112352 (PMC9699506; doi:10.3390/pharmaceutics14112352)
Supplement: Supplementary file 1 [file pharmaceutics-14-02352-s001.zip › pharmaceutics-1991848-supplementary.pdf]

SUPPLEMENTARY MATERIAL

Novel pullulan/gellan gum bilayer film as a vehicle for silibinin-loaded nanocapsules in topical treatment of atopic dermatitis

Mailine Gehrcke[1], Carolina Cristóvão Martins[2], Taíne de Bastos Brum[1], Lucas Saldanha da Rosa[3], Cristiane Luchese[2], Ethel Antunes Wilhelm[2], Fabio Zovico Maxnuck Soares[3], Letícia Cruz[1*]

[1] Laboratório de Tecnologia Farmacêutica, Programa de Pós-graduação em Ciências Farmacêuticas, Centro de Ciências da Saúde, Universidade Federal de Santa Maria, Santa Maria CEP 97105-900, RS, Brazil
[2] Laboratório de Pesquisa em Farmacologia Bioquímica – Centro de Ciências Químicas, Farmacêuticas e de Alimentos, Universidade Federal de Pelotas, Pelotas CEP 96010-900, RS, Brazil
[3] Laboratório de Biomateriais, Centro de Ciências da Saúde, Departamento de Odontologia Restauradora, Universidade Federal de Santa Maria, Santa Maria CEP 97015-372, RS, Brazil
* Correspondence: leticia.cruz@ufsm.br; Tel.: +55 55 32209373

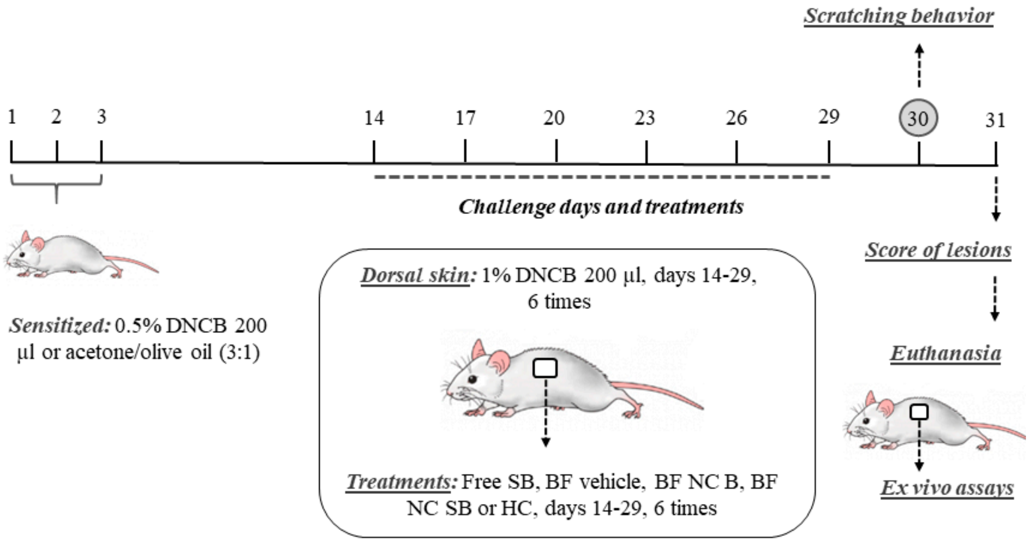

Figure S1. Schematic representation of the experimental design of *in vivo* study. DNCB (2, 4-dinitrochlorobenzene); Free SB (free silibinin solution); BF Vehicle (bilayer vehicle film); BF NC B (bilayer film containing unloaded nanocapsules); BF NC SB (bilayer film containing nanoencapsulated silibinin); HC (hydrocortisone ointment).
